# Pleistocene climate change and phylogeographic structure of the *Gymnocarpos przewalskii* (Caryophyllaceae) in the northwest China: Evidence from plastid DNA, ITS sequences, and Microsatellite

**DOI:** 10.1002/ece3.5113

**Published:** 2019-04-02

**Authors:** Shu‐wen Jia, Ming‐li Zhang

**Affiliations:** ^1^ Key Laboratory of Biogeography and Bioresource in Arid Land, Xinjiang Institute of Ecology and Geography Chinese Academy of Sciences Urumqi China; ^2^ University of Chinese Academy of Sciences Beijing China; ^3^ Institute of Botany Chinese Academy of Sciences Beijing China

**Keywords:** Genetic variation, *Gymnocarpos przewalskii*, Northwestern China, Pleistocene climate change

## Abstract

Northwestern China has a wealth of endemic species, which has been hypothesized to be affected by the complex paleoclimatic and paleogeographic history during Quaternary. In this paper, we used *Gymnocarpos przewalskii* as a model to address the evolutionary history and current population genetic structure of species in northwestern China. We employed two chloroplast DNA fragments (*rps*16 and *psb*B‐*psb*I), one nuclear DNA fragment (ITS), and simple sequence repeat (SSRs) to investigate the spatial genetic pattern of *G. przewalskii*. High genetic diversity (cpDNA: *h*_S_ = 0.330, *h*_T_ = 0.866; ITS: *h*_S_ = 0.458, *h*_T_ = 0.872) was identified in almost all populations, and most of the population have private haplotypes. Moreover, multimodal mismatch distributions were observed and estimates of Tajima's *D* and Fu's *FS* tests did not identify significantly departures from neutrality, indicating that recent expansion of *G. przewalskii *was rejected. Thus, we inferred that *G. przewalskii* survived generally in northwestern China during the Pleistocene. All data together support the genotypes of *G. przewalskii* into three groups, consistent with their respective geographical distributions in the western regions—Tarim Basin, the central regions—Hami Basin and Hexi Corridor, and the eastern regions—Alxa Desert and Wulate Prairie. Divergence among most lineages of *G. przewalskii* occurred in the Pleistocene, and the range of potential distributions is associated with glacial cycles. We concluded that climate oscillation during Pleistocene significantly affected the distribution of the species.

## 1 INTRODUCTION

Complex paleoclimatic and paleogeographic history has left a profound influence on the distribution and the spatial genetic structure of plant species in the Northern Hemisphere (Hewitt, 2000). Many geologic and climatologic reports prove that China also has been affected by this complexity (Guo et al., 2006; Li, Shu, Zhou, Zhao, & Zhang, 2004; Ma & Gao, 2004). In order to understand the degree of influence of history on the Chinese flora and fauna, a plethora of phylogeographic researches have focused on the Sino‐Japanese Floristic Region and Qinghai‐Tibet Plateau in recent decades (Li, Yue, Sun, & Qian, 2012; Li et al., 2013; Poudel et al., 2015; Ye, Zhu, Chen, Zhang, & Bu, 2014). In contrast, few such studies focusing on plant species from northwestern China have been reported (Gao, Meng, & Zhang, 2014; Zhang & Zhang, 2012a).

Northwestern China has experienced complex orogenesis and climate change. Following the tectonic uplift of the Qinghai‐Tibet Plateau, the Paratethys Ocean was compelled to retreat from Central Asia, and the beginnings of an arid climate emerged (Guo et al., 2002; van Hinsbergen et al., 2012; Zhang, Dong, Yu, & He, 2013). Over a lengthy period of successive growth of the Qinghai‐Tibet Plateau from mid‐Tertiary onwards, the climate of northwestern China was increasingly dry, because moist sea breezes from the Indian Ocean were obstructed, and the Pacific monsoon could not reach inland (Chen, Liu, Zhou, & Wang, 1999). In addition, glacial–interglacial cycle had further impacts on the climate of the region during the Quaternary. Both climatic oscillations and historical orogenesis have caused complex landscapes and drier climates in northwestern China. Duo to aridification and orogenic, a large number of deserts and mountains of northwestern China have emerged as effective geographical barriers, resulting in fragmentation of species’ distributions and limited gene flow between fragmentations. Thus, in the populations of many desert plants have higher total genetic diversity but lower within populations genetic diversity, and allopatric divergence has generally been found in desert plants, for example, *Ribes meyeri* (Xie & Zhang, 2013), *Hexinia polydichotoma* (Su, Zhang, & Cohen, 2012), *Helianthemum songaricum* (Su, Zhang, & Sanderson, 2011), *Nitraria sphaerocarpa* (Su & Zhang, 2013), and *Atraphaxis frutescens* (Xu & Zhang, 2015). Due to dry glacial–humid interglacial cycle in the Quaternary in these regions, many species experienced glacial contraction and postglacial expansion with corresponding climate cycle. Several glacial refugia have been found in Ili Valley, Tianshan Mountains, and Helan Mountains (Meng & Zhang, 2011; Shi & Zhang, 2015; Zhang & Zhang, 2012b; Zhang, Zhang, & Sanderson, 2013). Previous study also found that mountain ranges surrounding the Dzungarian Basin probably served as migration corridors for *Clematis sibirica* and the Loess Plateau was a dispersal corridor for *Lagochilus ilicifolius *during postglacial time (Meng & Zhang, 2011; Zhang & Zhang, 2012a). In the past few decades, although the phylogeography has developed rapidly in northwestern China, the evolutionary history of most plants in these regions is still poorly understood.

**Figure 1:**
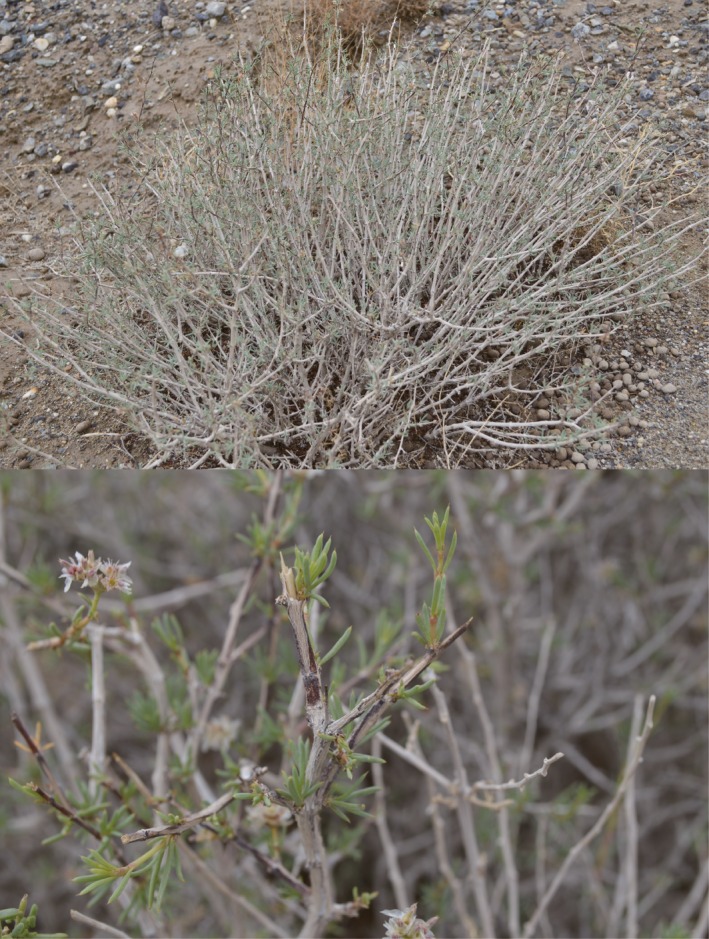
Gymnocarpos przewalskii

*Gymnocarpos przewalskii* (Figure 1) is a small shrublet distributed throughout the semi‐arid regions of northwestern China, from the westernmost end of the Tarim Basin, through the Hami Basin and the Hexi Corridor, to the Wulate Prairie at the easternmost. The plant has succulent‐mucronate leaves and a well‐developed root system, and shows an outstanding adaptation to arid regions. It has low dispersal capabilities, because both seeding and germination rates are low, and although it can propagate clonally, the dispersal of cloned offspring is very limited in scope and concentrated around the mother plant (Wang & Ma, 2007). Therefore, *G. przewalskii* can serve as a good model for spatial and temporal scale phylogeographic analysis in the region. Moreover, it is a protected plant of first conservation priority (Fu, 1992). Thus, the phylogeographic study of the species has important values, for both the origin and evolution of flora of the Chinese northwestern deserts, and plant protection. Previous phylogeographic studies of *G. przewalskii* (Ma, Zhang, & Sanderson, 2012) based on chloroplast genetic data alone showed the species to have high levels of genetic variation, especially in the western Tarim Basin, the Hami Basin, the Liuyuan region in western Gansu, and at the easternmost end of the distribution of the species. To explain the above genetic structure, a scenario that the four regions were glacial refugia for *G. przewalskii *was hypothesized (Ma et al., 2012). However, that study used only chloroplast DNA fragments, and therefore, the ability to investigate population dynamics and genetic structure of the species was limited.

In the present study, we employed both nuclear and chloroplast markers and simple sequence repeat (SSRs) to investigate the spatial genetic pattern of *G. przewalskii*. In angiosperm species, the chloroplast genome is maternally inherited from the ovary, while the nuclear genome is biparentally inherited from both pollen and ovary. The different patterns of inheritance of markers may reveal the complexity of gene flow because the distribution of genetic diversity estimated from different patterns of inheritance of markers may be tremendous difference (Petit et al., 2005). Thus, the two modes of inheritance allow us to examine the population structure from different perspectives. In this work, we aimed to address the following questions: (1) to reveal the evolutionary history and (2) explore current population genetic structure of *G. przewalskii* from in northwestern China.

## 2 MATERIALS AND METHODS

### 2.1 Sample collection

**Figure 2:**
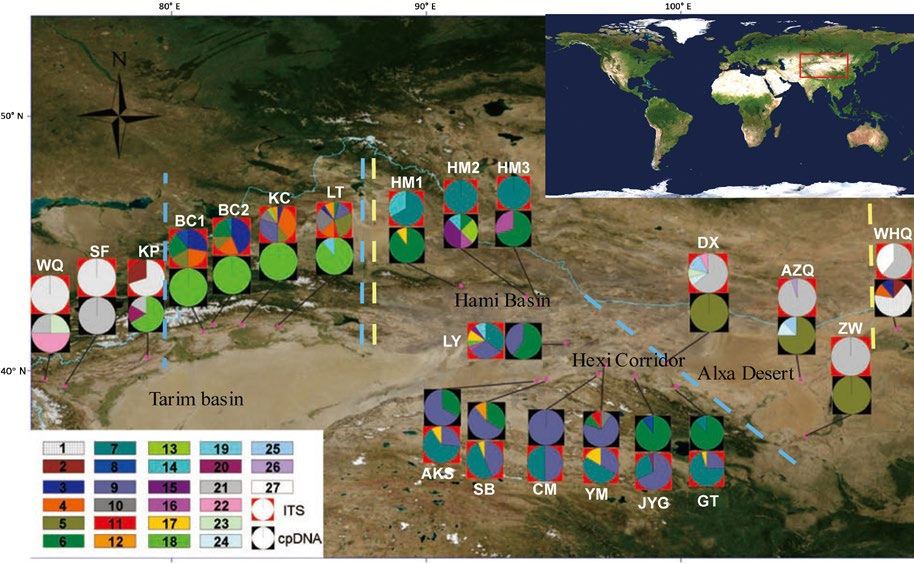
Geographical distributions in northwestern China for cpDNA and ITS haplotypes found in *G. przewalskii*. Red boxes represent ITS haplotypes; black boxes represent cpDNA haplotypes; color bars represent haplotypes for both ITS and cpDNA. Pie charts show the different haplotypes and their proportions in each population. Blue dotted lines represent groups defined by the results of SAMOVA of ITS. Yellow dotted lines represent groups defined by the results of phylogenetic trees of cpDNA

For the analysis of nuclear and chloroplast markers, samples of *G. przewalskii* were collected during field surveys from June to September 2013. A total of 194 individuals from 21 sites were sampled, representing nearly all known populations across most of the *G. przewalskii* range (*n* = 3–12 individuals per population; Figure 2, Table. 1). Geographical coordinates were taken at the point of sampling for each population using a global positioning system (GPS) unit.

**Table 1:** Sampling localities and sample sizes in cpDNA and ITS analysis

Region	Population/code	Latitude	Longitude	*N*
Tarim Basin	Wuqia, XJ/WQ	39.68	75.01	4
	Shufu, XJ/SF	39.42	75.83	11
	Keping, XJ/KP	40.55	79.067	5
	Baicheng1, XJ/BC1	41.55	81.25	10
	Baicheng2, XJ/BC2	41.85	81.633	10
	Kuche, XJ/KC	41.85	82.77	10
	Luntai, XJ/LT	41.77	84.23	11
Hami Basin	Hami1, XJ/HM1	43.333	91.383	12
	Hami2, XJ/HM2	42.894	93.937	10
	Hami3, XJ/HM3	42.595	94.452	10
Hexi Corridor	Akesai, GS/AKS	39.598	94.343	11
	Shubei, GS/SB	39.669	94.719	10
	Liuyuan, GS/LY	41.105	95.505	10
	Changma, GS/CM	39.9	96.786	3
	Yunmen, GS/YM	40.247	96.983	10
	Jiayuguan, GS/JYG	39.785	98.198	10
	Gaotai, GS/GT	39.383	99.834	10
Alxa Desert	Dingxin, GS/DX	40.322	99.462	7
	Alashanzuoqi, IM/AZQ	39.648	104.71	10
	Zhongwei, NX/ZW	37.434	104.919	10
Wulate Prairie	Wulatehouqi, IM/WHQ	41.67	108.5	10
	Total			194

For the analysis of SSRs, most of the samples were collected in 2013 and added a portion of the samples in field surveys in 2015. A total of 136 individuals from 17 sites were sampled (*n* = 8 individuals per population; Supporting information Table S1).

### 2.2 DNA extraction, amplification, and sequencing

Genomic DNA from a single *G. przewalskii* individual was extracted according to a modified CTAB protocol (Doyle & Doyle, 1987), and the quality of the DNA was tested using electrophoresis in 1% agarose gel.

For the nuclear and chloroplast markers, we used total DNA to amplify and sequence two cpDNA fragments, *rps16* (Chen et al., 2011) and *psbB‐psbH *(Xu, Abe, Sakai, Kanazawa, & Shimamoto, 2000), and one nuclear fragment, ITS (White, Bruns, Lee, & Taylor, 1990). The PCR amplification program followed the protocol: 94˚C for 2 min, followed 94°C by 28 cycles 45 s, annealing at 52°C for 45 s, 72°C for 60 s, and an additional extension in 72°C for 10 min. PCR products were purified with Purification Kits (Qiagen) and sequenced in both directions by ABI 3730 automated sequencer (Shanghai Bioengineering, Shanghai, China). DNA sequences were edited and assembled in Seqman (Lasergene, DNASTAR Inc., Madison, Wisconsin, USA), aligned with Clustal X version 1.81 (Thompson, Gibson, Plewniak, Jeanmougin, & Higgins, 1997), and then checked by eye. Double peak sites were present in sequences of some ITS fragments, indicating heterozygous genotypes. To obtain haplotypes from unphased genotypes, we used the Bayesian method of PHASE 2.1 (Stephens & Donnelly, 2003; Stephens, Smith, & Donnelly, 2001), implemented in DnaSP v5 software with default parameters (Librado & Rozas, 2009), and checked by eye using MEGA6.05 (Tamura, Stecher, Peterson, Filipski, & Kumar, 2013). In analysis of recombination, we used newly obtained ITS sequences. Recombination was tested within the ITS alignment of the *G. przewalskii* data set using the pairwise homoplasy index (Bruen, Philippe, & Bryant, 2006), implemented in the software SPLITSTREE v.4.11 (Huson & Bryant, 2006).

For the analysis of SSRs, microsatellite enrichment library was constructed according to the protocols reported by Jia, Liu, Han, Li, and Pan (2011), with minor modifications.

Microsatellite loci were screened by the SSRHunter software (Li & Wan, 2005). The redundant sequences were discarded manually. The primer pairs were designed for the microsatellite loci with suitable flanking sequences by NCBI primer blast. The primer pairs were initially screened against eight individuals to test for amplification and polymorphism. A M13‐tail (TGTAAAACGACGGCCAGT) was added to the forward primers of all the promising loci for fluorescent dye labeling (Schuelke, 2000). Amplifications were performed in 25 μl reactions containing 60–100 ng genomic DNA, 2.0 10× PCR buffer, 1.6 μl dNTPs (2.5 mmol/L), 2.0 μl MgCl_2_ (25 mmol/L), 0.1 μL forward primer (10 μmol/L), 0.4 μl reverse primer (10 μmol/L) and fluorescently labeled (FAM) M13 primer (10 μmol/L), and 0.2 U Taq polymerase. PCR cycling conditions were as follows: 5 min at 95°C, followed by 30 cycles of 30 s at 95°C, 45 s at 47–64°C, and 45 s at 72°C, an additional 8 cycles of 30 s at 95°C, 45 s at 53°C, and 45 s at 72°C, and a final 12‐min extension step at 72°C (Schuelke, 2000). The amplified fragments were analyzed with an ABI 3730XL (Shanghai Bioengineering, Shanghai, China). The resulting electropherograms were evaluated with GeneMapper 4.0 (Shanghai Bioengineering, Shanghai, China). In the enrichment library, 272 positive clones were sequenced. After discarding redundant sequences, 40 microsatellite sequences with suitable flanking regions were obtained for the designed primer pairs. Among the 40 tested markers, 17 loci were amplified successfully in *G. przewalskii*, and 12 loci were polymorphic (Supporting information Table S2). Six polymorphic loci were used to determine genetic diversity of 17 populations from *G. przewalskii*.

### 2.3 Analysis of nuclear and chloroplast markers

Haplotype number (*N*) was determined using DnaSP v5 (Librado & Rozas, 2009). Nucleotide diversity (*π*), haplotype diversity (*h*), Tajima's *D* (Tajima, 1989), and Fu's *FS* tests (Fu & Li, 1993) were estimated using ARLEQUIN version 3.1 (Excoffier, Laval, & Schneider, 2005). The spatial genetic structure of *G. przewalskii* was conducted using a spatial analysis of molecular variance (Dupanloup, Schneider, & Excoffier, 2002) implemented in the software SAMOVA 1.0. This program can define partitions (*K*) of populations that are maximally differentiated from each other without a priori assumptions about population groupings. The analysis was run for *K* = 2–20, starting from 100 random initial conditions for each run. *F*_CT_ values were given for every calculation. Finally, we obtained two independent *K* groupings with maximized *F*_CT_ values (Iwasaki, Aoki, Seo, & Murakami, 2012) for the cpDNA and ITS, respectively. For ITS data, differentiations among the groups defined by the SAMOVA analysis were estimated by AMOVA and pairwise *F*_ST_ values, implemented in ARLEQUIN 3.1(Excoffier et al., 2005). Due to the SAMOVA analysis cannot group cpDNA sequence, we performed AMOVA and pairwise *F*_ST_ analysis for cpDNA using groups defined by phylogenetic analyses.

PERMUT was used to estimate *h*_S_ (within population diversity), *h*_T_ (total gene diversity), *G*_ST _(coefficient of genetic variation over all populations), and *N*_ST_ (coefficient of genetic variation between haplotypes). Phylogeographical structure is present if *N*_ST_ is significantly higher than *G*_ST_ (Pons & Petit, 1996). The relationships among haplotypes were estimated using the median‐joining method (Bandelt, Forster, & Röhl, 1999), implemented in Network version 4.6.1.2.

Mantel tests were used to investigate patterns of isolation by distance (IBD), which test the correlation between the matrix of pairwise genetic distance (*F*_ST_) values and the matrix of geographical distances. The analyses were carried out in the Alleles In Space software (Miller, 2005).

Phylogenetic analyses were performed using maximum parsimony (MP) and Bayesian inference (BI) methods. Outgroups for the cpDNA analysis were *Silene noctiflora* (JF715056, complete genome) and *Silene atocioides *(EU314655, EU308518); outgroups for ITS were *Silene noctiflora *(FN821141), *Paronychia canariensis *(AJ310959)*,* and *Gymnocarpos decandrus *(KF850591). Outgroup sequences were downloaded from NCBI (http://www.ncbi.nlm.nih.gov/). The haplotypes from unphased ITS genotypes of *Silene noctiflora *were obtained using methods similar to those with *G. przewalskii*. Optimal models of DNA evolution for the data were inferred using the MrModelTest 2.3 program (Nylander, 2008). The best substitution model was GTR + G for both cpDNA and ITS according to the Akaike information criterion, and was applied in BI, and BEAST analyses. The MP analyses were conducted in PAUP 4.0b10 (Swofford, 2002), with 1,000 additional sequence replicates and branch‐swapping using TBR. Parsimony bootstrapping (PB) was calculated from 1,000 replicates using fast and stepwise addition of taxa. BI analysis was carried out using MrBayes version 3.0b4 (Huelsenbeck, Ronquist, & Hall, 2003), with 2,000,000 generations in two parallel runs, sampling trees at every 1,000th generation. The first 10% of sampled trees were treated as burn‐in and discarded.

To investigate approximate divergence times of *G. przewalskii, *we employed a Bayesian approach in the software BEAST version 1.8.1. An uncorrelated lognormal relaxed clock was used for clock models, and a constant size process was used for modeling speciation. The MCMC search was run for 70,000,000 and 80,000,000 generations for ITS and cpDNA, respectively, and sampled every 1,000 generations. Four independent Markov chains were used in this process. TRACER 1.5 (Rambaut & Drummond, 2009) was used to check the convergence of the MCMC chains. The maximum clade credibility (MCC) tree was generated in TreeAnnotator using the product method with a burn‐in of the first 10% of sampled trees. Due to the lack of specific substitution rates in *G. przewalskii*, we estimated the divergence time using published substitution rates. Based on the synonymous substitution rates for most angiosperm species of cpDNA genes (1.0 × 10^−9^ to 3.0 × 10^−9^ s/s/y) (Wolfe, Li, & Sharp, 1987), and substitution rates of plant ITS (3.46 × 10^−9^ to 10 × 10^−9^ s/s/y) (Richardson, Pennington, Pennington, & Hollingsworth, 2001), we followed Zhang, Zhang, et al. (2013) in using a normal prior distribution and set a mean of 2 × 10^−9^ s/s/y and a standard deviation of 6.08 × 10^−10^ s/s/y for cpDNA, and a mean of 6.73 × 10^−9^ s/s/y and a standard deviation of 1.99 × 10^−9^ s/s/y for ITS.

To investigate the demographic history of *G. przewalskii*, we employed mismatch distribution analysis (MDA) implemented in ARLEQUIN 3.1 (Excoffier et al., 2005). MDA represents the frequency distribution of pairwise differences among haplotypes; multimodal distributions are related to demographic decline or equilibrium, while unimodal pairwise mismatch distributions indicate that populations have experienced recent demographic expansion (Rogers & Harpending, 1992; Slatkin & Hudson, 1991). The Harpending raggedness index (HRag) and the sum of squared deviations (SSD) between observed and expected mismatch distributions were estimated with 1,000 parametric bootstrap replicates.

### 2.4 Analysis of SSRs

To estimate genetic diversity at population or locus level, we calculate several parameters including the numbers of alleles (*N*_A_), the number of effective alleles (*N*_E_), observed heterozygosity (*H*_O_), expected heterozygosity (*H*_E_), allelic fixation index (*F*), gene flow (*N*m), and Hardy–Weinberg equilibrium (HWE) by POPGENE 1.32 (Yeh, Yang, Boyle, Ye, & Mao, 1997); allelic richness (*A*_R_) was estimated by HP‐RARE 1.1 (Kalinowski, 2005).

To indicate the genetic relationship among populations, an UPGMA tree based on pairwise Nei's genetic distances was constructed by POPGENE 1.32 (Yeh et al., 1997). Population genetic relationship was further assessed by the Bayesian clustering analysis using STRUCTURE version 2.3.4 (Pritchard, Stephens, & Donnelly, 2000). The optimal number of subgroups was set from 2 to 16 (*K* = 2–16). For each *K *value, ten independent runs were conducted with a burn‐in period of 10,000 and 10,000 Markov Chain Monte Carlo (MCMC) generations.

To compare the genetic differentiation within individuals, within populations, among populations within the groups and among the groups, a hierarchical analysis of molecular variance (AMOVA) was carried using Arlequin 3.1(Excoffier et al., 2005). Mantel tests were also carried out in the Alleles In Space software (Miller, 2005).

### 2.5 Species distribution modeling

We performed species distribution modeling (SDM) to reconstruct the paleo‐ and current distributions for *G. przewalskii* through the maximum entropy algorithm as implemented in MAXENT v3.3 (Phillips, Anderson, & Schapire, 2006). MAXENT employs environmental data in conjunction with presence‐only data to estimate the probability distribution. Species occurrence data were taken from our field surveys and the online database of the Chinese Virtual Herbarium (CVH; http://www.cvh.org.cn/cms/). In total, 27 points were obtained to be used for SDM analyses (Supporting information Table S3). SDM predicted current distribution using climatic variables at 2.5‐min resolution, downloaded from the WorldClim database (www.worldclim.org). The nineteen bioclimatic variables were strongly correlated with each other, which could result in model overfitting (Graham, 2003). To avoid this defect, we computed Pearson’ s correlation coefficient between each pair of variables, implemented in IBM SPSS Statistics 16.0 (IBM, Armonk, NY, USA). The variables with correlation coefficient >0.8 were considered as highly correlated. Finally, the models included seven variables: annual mean temperature (BIO 1), mean diurnal temperature range (BIO 2), temperature seasonality (BIO 4), mean temperature of the driest quarter (BIO 9), annual mean precipitation (BIO 12), precipitation of the driest month (BIO 14), and precipitation seasonality (BIO 15) (Supporting information Table S4). We projected the model using default settings for all parameters. Model performance was evaluated using the area under the receiver operating characteristic curve (AUC) calculated by MAXENT.

The data set for the LGM climate was downloaded from the WorldClim database at a resolution of 2.5 min and projected using the species’ current bioclimatic niche onto past climate layers. To construct the LGM potential distributions, we projected the model in both general circulation models: the Community Climate System Model (CCSM; Collins et al., 2006) and the model for interdisciplinary research on climate (MIROC Version 3.2; Hasumi & Emori, 2004).

## 3 RESULTS

### 3.1 Recombination analysis of ITS data set

Recombination was not detected in the ITS alignment by the pairwise homoplasy index (*p* = 0.738). Lack of recombination allowed direct analysis of the ITS sequences.

### 3.2 Genetic diversity

For cpDNA, the data set comprised 176 cpDNA sequences, which collapsed into 25 haplotypes. Mean *h* and *π* estimates were 0.8442 and 0.008417, respectively. For ITS, the data set comprised 370 ITS sequences yielding 27 haplotypes; mean *h* and *π* estimates were 0.8477 and 0.008825, respectively. The detail results of sequence diversity for each population are shown in Table 2.

**Table 2:** Haplotype diversity (*h*) and nucleotide diversity (*π*) in each geographical population

Population	cpDNA haplotypes	*h *(*SD*)	*π* (*SD*)	ITS haplotypes	*h *(*SD*)	*π* (*SD*)
WQ	H21 H22^a^ H23^a^	0.8333 (0.2224)	0.003505 (0.002560)	D1	0	0
SF	H21	0	0	D1	0	0
KP	H18 H20^a^ H21	0.6000 (0.2152)	0.005654 (0.003528)	D1 D2^a^	0.4667 (0.1318)	0.000728 (0.000781)
BC1	H18	0	0	D3 D4 D5 D6 D8^a^	0.8301 (0.0409)	0.002715 (0.001853)
BC2	H18	0	0	D3 D4 D5 D6 D7	0.7667 (0.0797)	0.002429 (0.001715)
KC	H18	0	0	D3 D4 D5 D9 D10 D12 D13^a^	0.8000 (0.0611)	0.002797 (0.001884)
LT	H18 H19^a^	0.2222 (0.1662)	0.002414 (0.001535)	D3 D4 D5 D6 D9 D10 D11^a^ D12	0.8615 (0.0420)	0.003413 (0.002192)
HM1	H6 H17^a^	0.2000 (0.1541)	0.000137 (0.000208)	D7 D14^a^	0.4638 (0.0695)	0.000727 (0.000736)
HM2	H6 H13^a^ H14^a^ H15^a^ H16	0.8571 (0.1083)	0.013108 (0.007408)	D7	0	0
HM3	H6 H16	0.4667 (0.1318)	0.002536 (0.001580)	D7	0	0
AKS	H6 H9	0.5000 (0.1283)	0.003055 (0.001882)	D7 D9 D17	0.5368 (0.0904)	0.000923 (0.000858)
SB	H6 H9 H12^a^	0.6389 (0.1258)	0.004239 (0.002520)	D7 D9 D17	0.5917 (0.0675)	0.001032 (0.000939)
LY	H6 H9	0.5333 (0.0947)	0.003259 (0.001966)	D7 D9 D15^a^ D16^a^ D17 D18^a^ D19^a^	0.8000 (0.0611)	0.002128 (0.001533)
CM	H9	0	0	D7 D9	0.6000 (0.1291)	0.000940 (0.000991)
YM	H6 H9 H10^a^ H11^a^	0.5333 (0.1801)	0.004039 (0.002378)	D7 D9 D17	0.6471 (0.0691)	0.001199 (0.001029)
JYG	H6 H8	0.2000 (0.1541)	0.002444 (0.001531)	D7 D9 D20^a^	0.5421 (0.0763)	0.000949 (0.000878)
GT	H6 H7^a^	0.2500 (0.1802)	0.000853 (0.000683)	D7 D9 D17	0.4684 (0.1045)	0.000850 (0.000818)
DX	H5	0	0	D21 D22^a^ D23^a^ D24^a^ D25^a^	0.5934 (0.1438)	0.001826 (0.001403)
AZQ	H5 H24^a^ H25^a^	0.4643 (0.2000)	0.006765 (0.003948)	D21 D26^a^	0.1000 (0.0880)	0.000157 (0.000311)
ZW	H5	0	0	D21	0	0
WHQ	H1^a^ H2^a^ H3^a^ H4^a^	0.5833 (0.1833)	0.002793 (0.001737)	D21 D27^a^	0.5033 (0.0639)	0.000789 (0.000785)
Total		0.8442 (0.0139)	0.008417 (0.004216)		0.8477 (0.0107)	0.008825 (0.004692)

a Representative of the unique haplotypes.

Distribution of cpDNA and ITS haplotypes is shown in Figure 2 and Table 2. For cpDNA, 19 haplotypes were private, and the remainder were shared by two or more populations. Five haplotypes (H5, H6, H9, H18, and H21) were widely distributed. The haplotype H6 was the most widespread, distributed throughout the Hami Basin and Hexi Corridor; H9 was also distributed through the Hexi Corridor. The haplotypes H18 and H21 were found in the Tarim Basin, and H5 was distributed in the Alxa Desert. For ITS, 16 haplotypes were private. Two (D7, D9) were widely distributed, occurring throughout the Central Tarim Basin, Hami Basin, and Hexi Corridor.

In the SSR analysis, a total of 69 alleles were obtained across 17 populations, with an average of 11.5 alleles per locus. The number of effective alleles of six loci ranged from 2.1348 to 8.2979, with an average of 4.4909. The observed and expected heterozygosities range from 0.3407 to 0.7279 and from 0.5335 to 0.8827, respectively (Supporting information Table S5). The number of alleles per population ranged from 3.1667 to 4.6667, with an average of 3.8333. The observed and expected heterozygosities range from 0.3750 to 0.7292 and 0.4917 to 0.7139, respectively. The allele abundance ranged from 2.56 to 3.69. Detailed polymorphic information is shown in Table 3.

**Table 3:** Genetic diversity among the populations of *G. przewalskii *by SSRs analysis

Population	*N*_A_	*N*_E_	*A*_R_	*H*_O_	*H*_E_	HWE
WQ	3.5000	2.7454	3.10	0.6875	0.6597	0.0009
SF	4.1667	2.9112	3.21	0.5417	0.6014	0.2430
WS	3.3333	2.5255	2.89	0.4375	0.5431	0.0958
BC1	4.1667	2.9451	3.34	0.5000	0.6222	0.1787
BC2	4.3333	2.7507	3.38	0.5208	0.6375	0.5215
KC	3.3333	2.3413	2.83	0.6250	0.5639	0.7696
LT	3.6667	2.7386	3.15	0.7083	0.6319	0.1565
HM1	3.5000	2.5199	2.96	0.5625	0.6014	0.1462
HM3	4.3333	2.7466	3.31	0.6964	0.6350	0.0830
AKS	4.6667	3.3433	3.69	0.7292	0.6931	0.1693
SB	3.1667	2.0432	2.58	0.3750	0.5194	0.1675
LY	3.1667	2.0047	2.56	0.3750	0.4917	0.0362
CM	4.6667	3.2155	3.65	0.5417	0.7139	0.0702
JYG	3.8333	2.6864	3.13	0.5417	0.6111	0.9006
GT	4.3333	3.2859	3.56	0.5833	0.6542	0.5383
ZW	4.1667	2.9799	3.48	0.4970	0.6620	0.0586
AZQ	3.8333	2.6754	3.17	0.6458	0.6306	0.0583
Average	3.8922	2.7329	3.18	0.5628	0.6160	0.2467

Notes

*H*_o_: mean observed heterozygosity, *H*_e_: mean expected heterozygosity, *A*_r_: allelic richness, HWE: Hardy–Weinberg equilibrium; *N_A:_* mean number of alleles per population, *N_E:_* mean number of effective alleles.

### 3.3 Population structure

Estimates of average within population diversity and total diversity for both cpDNA and ITS were very high in *G. przewalskii *(cpDNA: *h*_S_ = 0.330, *h*_T_ = 0.866; ITS: *h*_S_ = 0.458, *h*_T_ = 0.872). For ITS data, *N*_ST_ (0.873) was significantly larger than *G*_ST_ (0.474) (*p* = 0.005 < 0.01), indicating the presence of significant phylogeographic structure across the species’ range. In contrast, we did not observe *N*_ST_ (0.700) significantly larger than *G*_ST_ (0.619) in the cpDNA data.

For SAMOVA results of ITS, populations were divided into four groups (GITS1, Western Tarim Basin: WQ, SF, and KP; GITS2, Central Tarim Basin: BC1, BC2, KC, and LT; GITS3, Hami Basin + Hexi Corridor: HM1, HM2, HM3, AKS, SB, LY, CM, YM, JYG, and GT; and GITS4, Alxa Desert + Wulate Prairie: DX, AZQ, ZW, and WHQ) as *F*_CT_ reached the maximum value (*F*_CT_ = 0.89225). The fixation index *F*_ST _by AMOVA and pairwise difference (Tables 4, 5) of ITS indicates that the greatest percentage (ITS: *F*_ST_ = 89.22%) can be explained by among‐group variation. The SAMOVA analyses using cpDNA indicated that a grouping structure was not present, because the *K* values got higher as *F*_CT_ got higher.

**Table 4:** Results of analysis of molecular variance of *G. Przawalskii* for three/four groups based on cpDNA/ITS sequence data

Source of variation cpDNA/ITS	*df*	Sum of squares	Variance components	Percentage of variation
Among groups	2/3	433.112/887.941	4.39574 Va/3.61117 Va	50.19/89.22
Among populations within groups	18/17	403.216/25.073	2.47004 Vb/0.06344 Vb	28.20/1.57
Within populations	155/347	293.433/129.313	1.89312 Vc/0.37266 Vc	21.61/9.21
Total	175/367	1,129.761/1,042.326	8.7588/4.04727	
Fixation Indices *F*_SC_: 0.56611/0.14547 *F*_ST_: 0.78386/0.90792 *F*_CT_: 0.50186/0.89225				

**Table 5:** The results of pairwise difference among four groups (defined by the results of SAMOVA for ITS) of ITS data

	GITS1	GITS2	GITS3	GITS4
GITS1	0.00000			
GITS2	0.74172^a^	0.00000		
GITS3	0.88551^a^	0.48540^a^	0.00000	
GITS4	0.97747^a^	0.90312^a^	0.95384^a^	0.00000

a Representative of the significant *F*_ST_ *p*‐value (*p* < 0.05).

**Figure 3:**
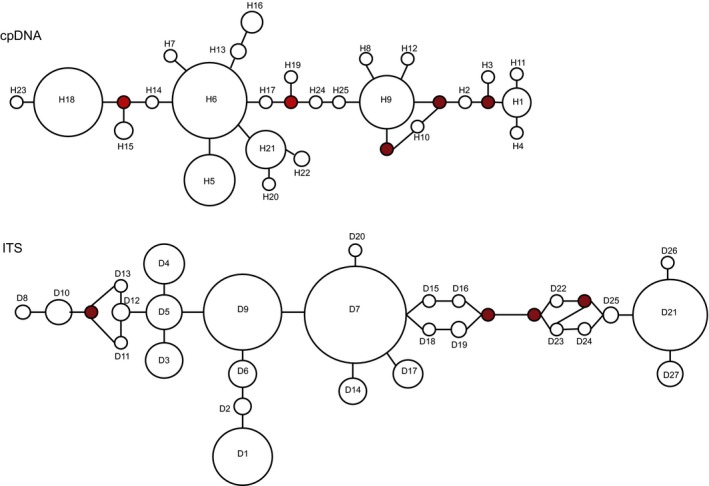
The haplotype networks in *G. przewalskii*. The red circle represents a hypothetical haplotype with another mutational step between real haplotypes. Circle sizes are proportional to haplotype frequencies in all samples

The Mantel tests of both cpDNA and ITS showed a significant correlation between genetic distance and geographical distance matrices (cpDNA: *r* = 0.260; *p* = 0.005 < 0.01; ITS: *r* = 0.505; *p* = 0.001 < 0.01), suggesting strong isolation by distance. The MJ Network illustrates the complex genealogical relationships among haplotypes found for cpDNA and ITS (Figure 3). The most ancestral haplotypes are expected to located at central position within a haplotype network, geographically widespread and consequently have more mutational connections to other haplotypes. Thus, H6, D7, and D9 may be the most ancestral haplotypes for the cpDNA and ITS data set, respectively.

**Figure 4:**
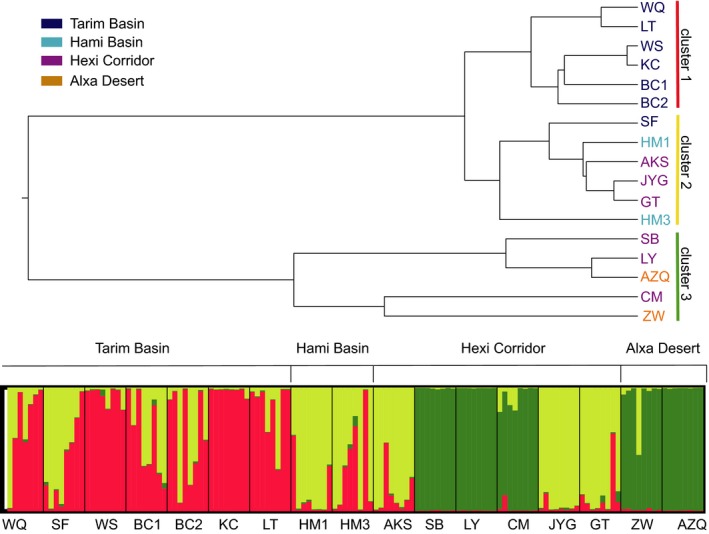
Above, UPGMA dendrogram between the 17 populations of *G. przewalskii* based on genetic distance; below, spatial genetic structure of *G. przewalskii* using Bayesian assignment probability analysis. Colors of bar represent clustering of populations

In analysis of SSRs, according to the Nei's genetic similarity coefficients and genetic distance, the relationship between populations SB, LY, CM, ZW, and AZQ is closest (Supporting information Table S6). The UPGMA tree showed that the 17 populations could be divided into three groups (Figure 4): The first group consists of populations from the Tarim Basin, including WQ, WS, BC1, BC2, KC, and LT; the second group consists of the Hexi Corridor and the Hami Basin, including SF, HM1, HM3, AKS, JYG, and GT; the third group consists of the Hexi Corridor and Alxa Desert populations, including SB, LY, CM, ZW, and AZQ. According to the STRUCTURE analysis, when the *K* value is equal to 3, the Deltak value is the largest (Supporting information Figure S1). Therefore, Bayesian clustering approach revealed that there are three groups in the population of *G. przewalskii* (Figure 4). The Bayesian approach showed a similar grouping pattern with that displayed by the UPGMA tree. The AMOVA results (Table 6) show that the maximum variation of the population of *G. przewalskii* occurred within individuals was 72.39304%, while 15.22903% of variation occurred among groups. Mantel tests showed that geographical isolation was significantly associated with genetic distance (*r = *0.1362; *p* = 0.0009) in all population of *G. przewalskii*. The geographical isolation and genetic distance were also significantly correlated with the populations of *G. przewalskii* in the Tarim Basin (*r* = 0.1168; *p* = 0.0169).

**Table 6:** The AMOVA results of *G. przewalskii *by SSRs analysis

Source of variation	Sum of squares	Variance components	Percentage variation
Among groups	71.629	0.35491	15.22903
Among populations within groups	54.617	0.11726	5.03158
Among individuals within populations	240.83	0.17121	7.34635
Within individuals	229	1.68712	72.39304
Total	596.076	2.3305	

### 3.4 Phylogenetic analyses and estimate of divergence time

**Figure 5:**
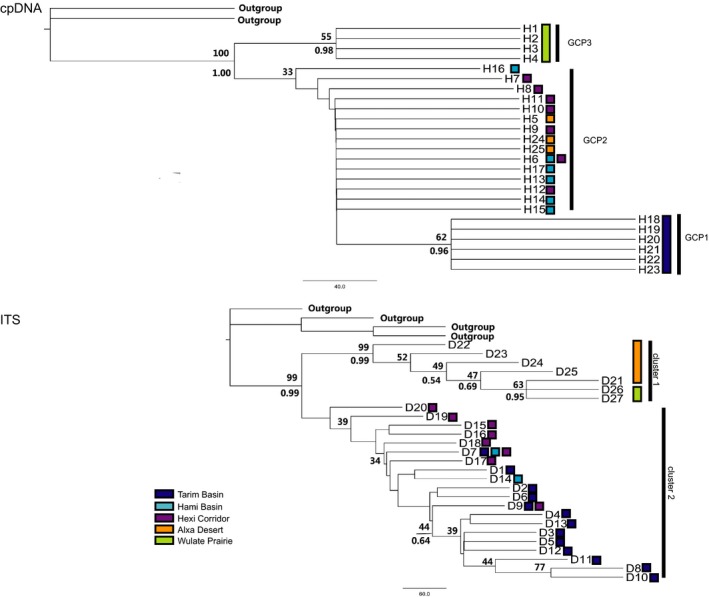
The phylogenetic relationships among *G. przewalskii* species based on both cpDNA and ITS sequences. Numbers above branches are support values from bootstrap resampling and Bayesian inference

The MP tree and Bayesian tree are highly similar, only differing in minor aspects, so we here employed the MP tree (Figure 5) as an example. In these trees, topologic structures are related to the geographical distribution of haplotypes. The MP cpDNA tree is comprised of three major clades (GCP1: Tarim Basin, GCP2: Hami Basin + Hexi Corridor + Alxa Desert, and GCP3: Wulate Prairie). The fixation index *F*_ST _by AMOVA and pairwise difference (Tables 4, 7) of cpDNA indicates that the greatest percentage (cpDNA: *F*_ST_ = 50.19%) can be explained by among‐group variation. The MP ITS tree is comprised of two major clusters (Tarim Basin + Hami Basin + Hexi Corridor and Alxa Desert + Wulate Prairie).

**Table 7:** The results of pairwise difference among three groups of cpDNA data

	GCP1	GCP2	GCP3
GCP1	0.00000		
GCP2	0.42131^a^	0.00000	
GCP3	0.87023^a^	0.69283^a^	0.00000

a Representative of the significant *F*_ST_ *p*‐value (*p* < 0.05). AL: Alxa Desert.

**Figure 6:**
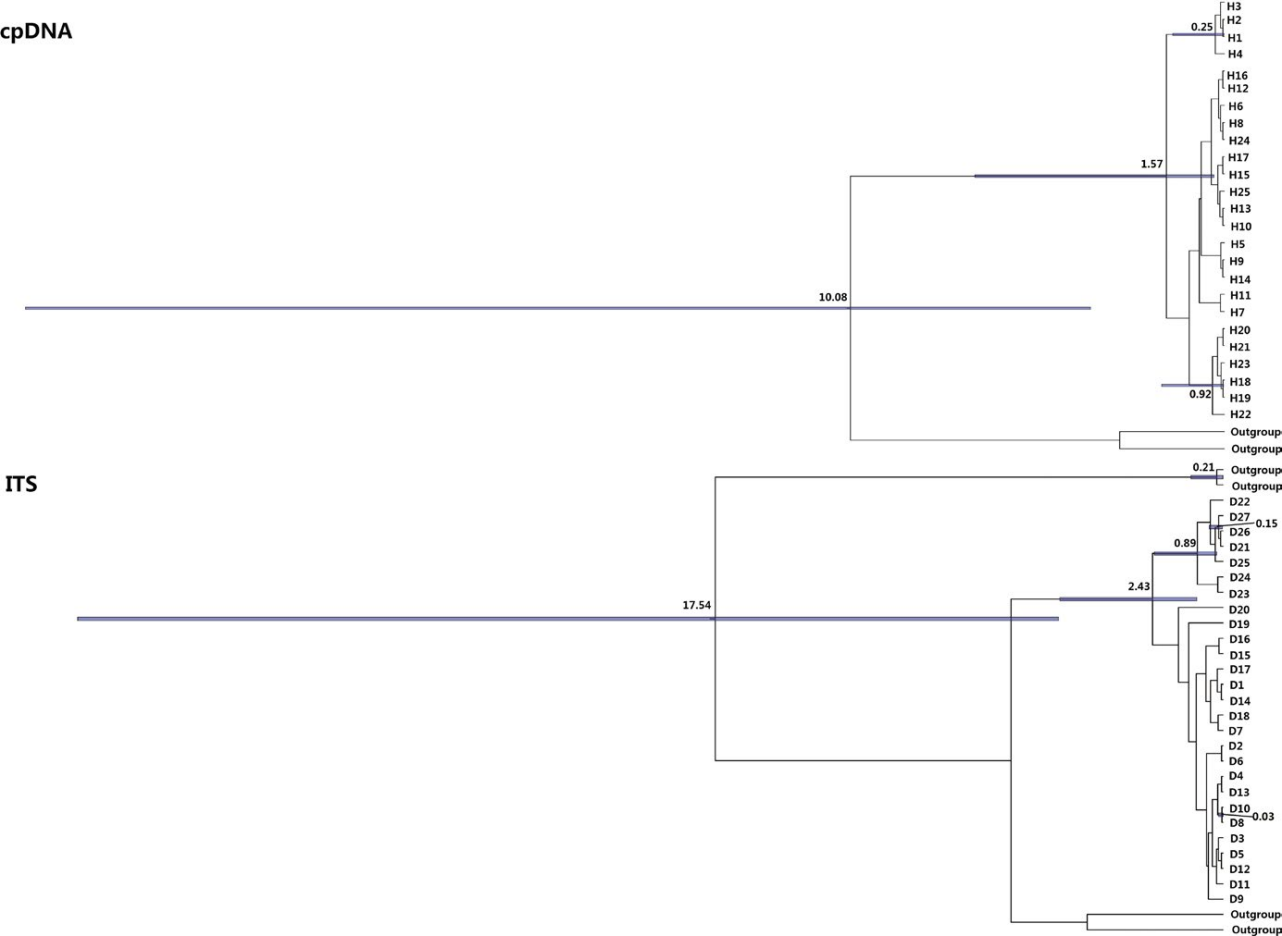
Divergence dating (Ma BP.) of both cpDNA and ITS haplotypes in *G. przewalskii* based on coalescence analysis

The results of BEAST for both ITS and cpDNA data showed that divergence between most lineages of *G. przewalskii* occurred in the Pleistocene (Figure 6).

**Figure 7:**
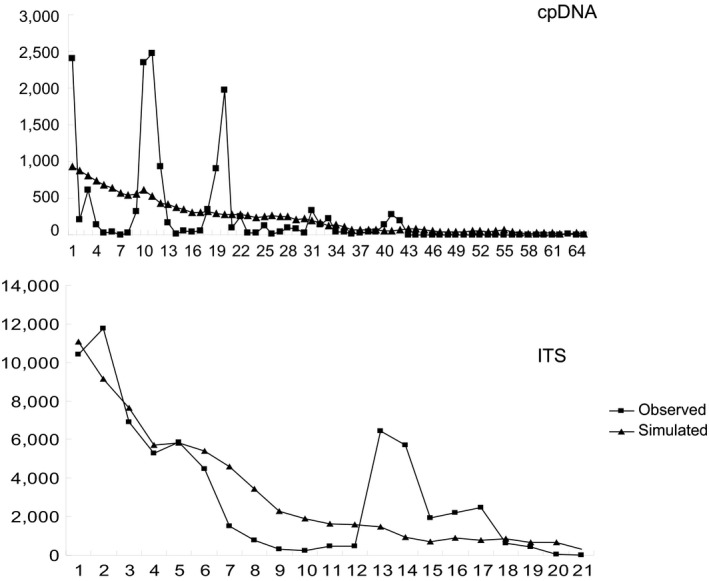
Mismatch distributions based on the cpDNA sequences and ITS sequences

Estimates of neutrality tests did not identify significantly departures from neutrality, with *p*‐values of the whole data above 0.05 (Table 8). In the mismatch distribution analysis (Figure 7), multimodal mismatch distribution was observed in both cpDNA data and ITS data set.

**Table 8:** Results of neutrality tests and mismatch distribution analysis for both cpDNA and ITS

	Hrag (*p* value)	SSD (*p* value)	Fu's *F*_s_ (*p* value)	Tajima's *D* (*p* value)
cpDNA	0.07538 (0.00000)	0.06871 (0.00000)	4.57825 (0.88900)	−0.72706 (0.26500)
ITS	0.02021 (0.90000)	0.01865 (0.80000)	−0.88993 (0.54800)	1.58183 (0.92100)

### 3.5 Species distribution modeling

**Figure 8:**
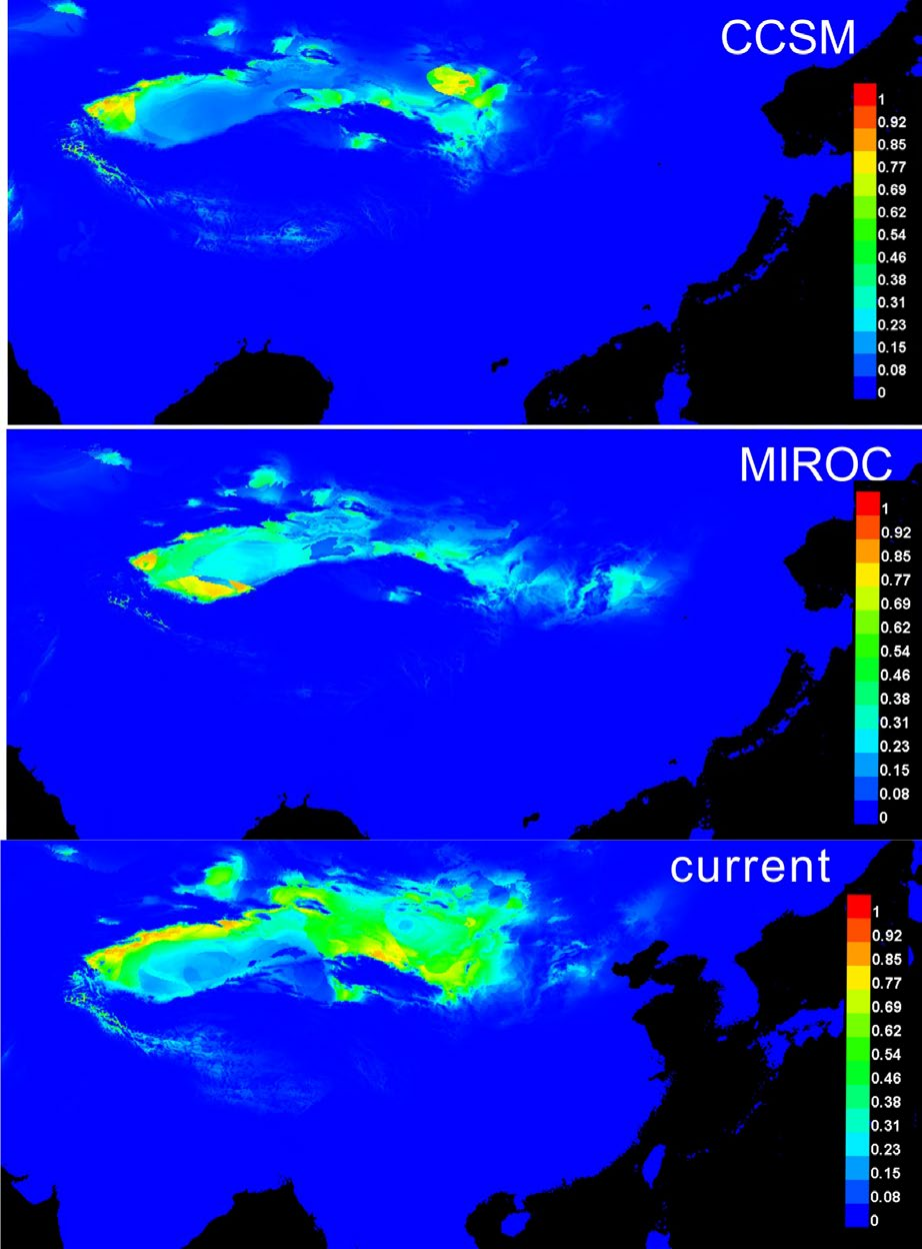
Modeling distribution of *G. przewalskii *onto the LGM and current conditions

For *G. przewalskii*, the average value of AUC is very high (AUC > 0.90), indicating that the SDM developed under the current climate conditions accurately predicted the distribution of the species. The averaged distributions for the LGM and current conditions predicted by this model are shown in Figure 8.

The present potential distribution of high support (0.6–1) for *G. przewalskii *predicted by MAXENT is basically consistent with the actually observed geographical distribution of the species. The illustration shows that the most suitable climatic conditions are concentrated in the northern edges of the Tarim Basin, Hami Basin, Hexi Corridor, and Alxa Desert.

The LGM potential distribution of the species was predicted by CCSM and MIROC models. Compared with the present potential distribution, the LGM distribution showed a general range contraction. Both CCSM and MIROC models showed that all these suitable areas were isolated from each other. Overall, the range of potential distributions is associated with glacial cycles; the distribution range contracted under glacial conditions and expanded during interglacials.

## 4 DISCUSSION

### 4.1 Genetic diversity in *G. przewalskii*

The cpDNA haplotype diversity (Tables 2, 3) estimated for *G. przewalskii* (*h*_T_ = 0.866) in northwestern China was found to be high compared to the average value (*h*_T_ = 0.670) for other plants (Petit et al., 2005). Similarly, the ITS haplotypes diversity (*h*_T_ = 0.872) was found to be high compared to several other plants occurring in adjacent regions (e. g., *Clematis sibirica* and *C. songorica*, *h*_T_ = 0.4963 and 0.3612, Zhang, Zhang, et al., 2013). High level of total diversity has been detected in several other species in northwestern China (e. g., *Ribes meyeri*, *h*_T_ = 0.857, Xie & Zhang, 2013; *Atraphaxis frutescens*, *h*_T_ = 0.858, Xu & Zhang, 2015; *Hexinia polydichotoma*, *h*_T_ = 0.739, Su et al., 2012; *Lagochilus ilicifolius*, *h*_T_ = 0.925, Meng & Zhang, 2011; *Calligonum calliphysa*, *h*_T_ = 0.886, Wen, Xu, Zhang, & Feng, 2015). Considering that the divergence of haplotypes of these species occurred during the Pleistocene, the high total genetic diversity detected in these taxa might reflect the fragmentation of habitats caused by aridity in the region, which limits gene flow and leads to rapid genetic divergence among different fragmentations and local adaptation (Su et al., 2012; Wen et al., 2015; Xie & Zhang, 2013). Our results of divergence time (Figure 6), mantel tests (cpDNA: *r* = 0.260; *p* = 0.005 < 0.01; ITS: *r* = 0.505; *p* = 0.001 < 0.01), pairwise difference (Tables 5, 7), and AMOVA (Table 4) analysis support the above hypothesis. According to floristic division of seed plants in China (Wu, Sun, Zhou, Li, & Peng, 2010), the distribution region of *G. przewalski *belongs to different regions of flora. Population of Tarim Basin and Hami Basin belong to sub‐Kawasya region, while the other population belong to the southwest Mongolia subregion (Wu et al., 2010), indicating that climate and vegetation types in two subregions are differences. The differences in these environments may have an impact on the genetic diversity of *G. przewalskii*. Moreover, *G. przewalski* represents the relicts of Tethyan ancestor. The high genetic diversity of the species might reflect the accumulation of mutations over time (Ma et al., 2012).

Compared to other species in this area, *G. przewalskii* has relatively high diversity (cpDNA: *h*_S_ = 0.330; ITS: *h*_S_ = 0.458) within most population (*Calligonum calliphysa*, cpDNA, *h*_s = _0.279, Wen et al., 2015; *Atraphaxis frutescens*, cpDNA, *h*_s_ = 0.092, Xu & Zhang, 2015). This result is consistent with the microsatellite, and the majority of the population have a moderate value of *H *(*H*_E_ = 0.6160, *H*_O_ *= *0.5628) compared to the average value (*H*_E_ = 0.61, *H*_O_ *= *0.58) for other plants (Nybom, 2004). The breeding methods and plant life of the species may contribute to high diversity within population. *G. przewalskii* is facultative clones that have sexual reproduction and cloning (Wang & Ma, 2007). The genetic diversity of these plants is closely related to the proportion of sexual reproduction in their reproductive systems, since sexual reproduction can increase genetic diversity within the population and reduce genetic differences among populations (Kirsten, Dawes, & Cochrane, 1998). The results of *HWE* show that most of the population of *G. przewalskii* did not deviate significantly from *HWE*, indicating random mating population (Table 3). In addition, the genetic diversity among the populations within group was the smallest, and the genetic variation among individuals was the largest, indicating that individuals within the group may be free to mate. Previous physiology studies have shown that *G. przewalskii* is mainly sexually propagated as cross‐pollination plants (Li, Tang, & Fu, 2016). Moreover, the seeds of the species have a strong drought resistance. Under drought stress, ungerminated seeds enter a dormant state and germinate quickly after stress. Therefore, the species have formed a mechanism of rapid and effective use of limited water to achieve reproductive (Yang, Liang, Chai, & Xue, 2017). The high genetic diversity of *G. przewalskii* may be due to the sexual reproduction of the individual within the group. The plant life also has an important effect on *h*_T_ and *h*_s_ of plants. Long‐lived plants may be less affected by genetic drift and are more likely to retain genotypes (Lowe, Boshier, Ward, Bacles, & Navarro, 2005). Aparicio, Hampe, Fernández‐Carrillo, and Albaladejo (2012) also show that the diversity of plants with high longevity is high. As the *G. przewalskii* is a small shrublet, long life may maintain high genetic diversity.

The strong phylogeographic structure among the population of *G. przewalskii *observed here was largely due to the few haplotypes shared among regions. The phylogenetic tree of cpDNA and STRUCTURE analysis of SSRs together support that the genotypes of Tarim Basin of *G. przewalskii* are a separate group (Figures 2, 4, 5). According to grouping strategy of SAMOVA of ITS, the GITS1 and GITS2 represent the Tarim group. The analysis of mantel tests and AMOVA indicated that there were huge genetic differences between Tarim and other regions. The phylogenetic and median‐joining tree of ITS support the separation of haplotypes of GITS4 (Alxa Desert + Wulate Prairie) from GITS3 (Hami Basin + Hexi Corridor). And almost no haplotypes were shared among the Tarim Basin, Hami Basin + Hexi Corridor, and Alxa Desert + Wulate Prairie (Figures 2, 3, 5, Table 2). Therefore, based on the available data, the genotypes of this species were roughly divided into three groups. The lack of shared cpDNA or ITS haplotypes among these three discrete groups indicated that there has been little gene flow among the groups since their formation, leading to the accumulation of haplotypes differences. Moreover, each of the three groups has its own distribution and habitat (Figure 2), which could also be responsible for the high genetic differentiation among groups. The Tarim Basin is surrounded by mountains on three sides, the Pamir Plateau on the west, the Tianshan Mountains on the north, and the Kunlun and Altun Mountains on the south, forming a relatively closed environment and preventing gene flow with other regions. Badain Jaran Desert and Tengger Desert are effective geographical barriers for population of Hami Basin + Hexi Corridor and Alxa Desert + Wulate Prairie. In addition, the weak dispersability of *G. przewalskii *seeds also have contributed to the large genetic distances and limited gene flow among groups (Ma & Zhang, 2012; Ma et al., 2012).

### 4.2 Phylogeographic history of *G. przewalskii*

Previous evolutionary studies have shown the aridification since mid‐late Miocene significantly affected the diversification of *Gymnocarpos* in the arid regions from North Africa to Central Asia, and intraspecific diversification of *G. przewalskii* mostly occurred during the Pleistocene (Jia, Zhang, Raab‐Straube, & Thulin, 2016). Our estimates of haplotype divergence of *G. przewalskii are* consistent with previous studies, suggesting that the Quaternary climate fluctuations and aridification have important influence on the current geographical distribution of the species. Drastically increased aridity occurred in northwestern China during the Pleistocene, leading to expansion of deserts in these regions. For example, the Taklamakan Desert greatly enlarged after the early Pleistocene (Sun, 2002; Zhang & Men, 2002). The initial Gurbantunggut Desert was present at the mid‐Pleistocene (Fang et al., 2002), the Badain Jaran‐Tengger Desert greatly enlarged during the mid‐Pleistocene (Yang, Fang, Dong, Peng, & Li, 2006), and the sand desert landscape developed in the Ulan Buh Desert in late‐Pleistocene (Li et al., 2014). Although *G. przewalskii* is an arid‐adapted species, moderate moisture is essential for its growth and reproduction. The edge of oases provides many habitats with moderate moisture (Xu & Zhang, 2015). Because of this reason, when the desert expanded, the gene flow caused by pollen and seeds was limited, resulting in the increase in genetic variation.

Coalescent theory predicts that ancient haplotypes will occupy interior nodes of a haplotype network and be geographically widespread, whereas the recent haplotypes will occupy at the tips of the haplotype network and be geographically local distribution (Schaal, Hayworth, Oseni, Rauscher, & Smith, 1998). In this study, most haplotypes from Hami Basin + Hexi Corridor are the interior haplotypes, especially H6, H9, D7, and D9. The four are the most interior and widespread haplotypes, and can be considered as ancestral haplotypes. In contrast, haplotypes from other regions lie on the edge of the haplotype network. Within the entire network, many haplotypes of Tarim Basin and Alxa Desert + Wulate Prairie group are derived. This is also suggested by phylogenetic tree. Many earliest diverging haplotypes in *G. przewalskii* are found in Hami Basin + Hexi Corridor (Figure 5), suggesting that these regions may be relatively ancient distributions of *G. przewalskii*. Thus, we speculate on a hypothesis that *G. przewalskii* would have the opportunity to occupy new areas such as Tarim Basin and Alxa Desert + Wulate Prairie during the Pliocene to the early to mid‐Pleistocene. Subsequent glacial–interglacial cycles are likely to have resulted in gradual isolation and divergence of haplotypes in Tarim Basin and Alxa Desert + Wulate Prairie. However, due to the limited data, further research is needed.

Biological refugia are relatively stable areas where a species is predicted to have had persistent survival throughout climatic fluctuations during the Pleistocene. According to Petit et al. (2003), plant populations that have high levels of genetic diversity and unique haplotypes indicate that their associated areas may have served as glacial refugia. Previous studies of *G. przewalskii* based on cpDNA data alone have suggested four independent glacial refugia for the species (the western Tarim Basin, Hami Basin, western Gansu, and the easternmost part of this species distribution area), because of high levels of genetic variation, unique haplotypes, and ancestral haplotypes in these four regions (Ma et al., 2012).

In present study, combining all data sets, we observed that almost all populations have high levels of genetic diversity, and 14 populations distributed in each group have unique haplotypes (Tables 2, 3). Thus, we observed neither hot spots nor cold spots, and could not find clines of genetic diversity from hypothetical refugial groups, or hypothetical recolonized groups. Furthermore, mismatch distribution analysis and the neutrality tests do not agree with a recent expansion (Figure 7, Table 8). Considering the characteristics of haplotype distribution and climate change process during the evolution of *G. przewalskii*, the Tarim Basin, the Hami Basin, the Hexi Corridor, the Alxa Desert, and the Wulate Prairie can be deduced as glacial refugia for species and localized range expansion from these refugia during the interglacial.

The distribution areas of *G.przewalskii* have a wealth of mountains that have complicated topology. The species in the Tarim Basin and the Hami Basin are mainly distributed along the southern Tianshan Mountains. The species in Hexi Corridor is mainly distributed along the northern Qilian Mountains. The species in Wulate Prairie is mainly distributed along the Yin Mountain. Populations of the species are distributed in scattered patchiness and mainly grow along foothills. Areas of complicated topography might provide suitable microenvironment in relative stability and survived there during severe climatic oscillations (Rull, 2009). In addition, there were mountains that do not exceed 2000 meters in northwestern China without ice cover during the Pleistocene. The foothills that locate between the desert and the mountains were characterized by a relatively mild Pleistocene climate. Thus, we hypothesized that there are many patch‐like stable microenvironment in the distribution of *G. przewalskii* so that the species can survive during climatic oscillations (Rull, Schubert, & Aravena, 1988). Thus, plant grown in microenvironment did not undergo large‐scale recolonization during interglacial, but rather a process of increasing the sizes of occupancy of its geographical distribution (Rull, 2009; Ye et al., 2014).

## CONFLICT OF INTEREST

None declared.

## AUTHORS’ CONTRIBUTION

M. Z. conceived and designed the experiments. S. J. collected samples, performed the experiments, and analyzed the data. M. Z. and S. J. wrote, edited, and reviewed the MS.

## Data Availability

The plastid DNA, ITS, and microsatellite sequences reported in this study were deposited in GenBank under accession numbers MH888219 ‐ MH888258 and MH917953 ‐ MH917999. The microsatellite loci data are available from the Dryad Digital Repository: https://doi.org/10.5061/dryad.834g5n6
