# Supplementary material for: Pleistocene climate change and phylogeographic structure of the Gymnocarpos przewalskii (Caryophyllaceae) in the northwest China: Evidence from plastid DNA, ITS sequences, and Microsatellite
Source: Ecol Evol. 2019 Apr 2;9(9):5219–35. doi: 10.1002/ece3.5113 (PMC6509395; doi:10.1002/ece3.5113)
Supplement: Supplementary file 2 [file ECE3-9-5219-s002.doc]

Table S1 Sampling localities and sample sizes in SSRs analysis.

| Region | Population/code | Latitude | Longitude | *N* |
| --- | --- | --- | --- | --- |
| Tarim Basin | Wuqia, XJ/WQ | 39.68 | 75.01 | 8 |
|  | Shufu, XJ/SF | 39.42 | 75.83 | 8 |
|  | Wensu, XJ/WS | 41.58 | 80.65 | 8 |
|  | Baicheng1, XJ/BC1 | 41.55 | 81.25 | 8 |
|  | Baicheng2, XJ/BC2 | 41.85 | 81.633 | 8 |
|  | Kuche, XJ/KC | 41.85 | 82.77 | 8 |
|  | Luntai, XJ/LT | 41.77 | 84.23 | 8 |
| Hami Basin | Hami, XJ/HM1 | 43.333 | 91.383 | 8 |
|  | Hnmi, XJ/HM3 | 42.595 | 94.452 | 8 |
| Hexi Corridor | Akesai, GS/AKS | 39.598 | 94.343 | 8 |
|  | Shubei, GS/SB | 39.669 | 94.719 | 8 |
|  | Liuyuan, GS/LY | 41.105 | 95.505 | 8 |
|  | Changma, GS/CM | 39.9 | 96.786 | 8 |
|  | Jiayuguan, GS/JYG | 39.785 | 98.198 | 8 |
|  | Gaotai, GS/GT | 39.383 | 99.834 | 8 |
| Alxa Desert | Alashanzuoqi, IM/AZQ | 39.648 | 104.71 | 8 |
|  | Zhongwei, NX/ZW | 37.434 | 104.919 | 8 |
|  | Total |  |  | 136 |

Table S2 Forty candidate primers were designed using blast primer of GenBank.

| Primer | Primer sequence （5 ` -3 ` ） | Primer | Primer sequence （5 ` -3 ` ） |
| --- | --- | --- | --- |
| Lgm 1 | GGC TTT GAC TGA TGG CAT GG | Lgm 21 | TGT ATA CCC CTC CTT GGT GAT T |
|  | CCA TTC ATC TAC TGT GAA GGA AAA T |  | AGA AAA GAC CAA CCA AAG CGT |
| Lgm 2 | CCG GTA GTC CGT GAC TTC TTT | Lgm 22 | AGA GTT CTA GAT AGG AGA |
|  | GGA GAA GGA TGA ACA ACT TGA AGA |  | GAT CTT CCC TAT AAA TAC |
| Lgm 3 | ATC ACC TAC AAC AAT AAC AAC CGT | Lgm 23 | CCG GCT AGG CTA CCT AAC TT |
|  | CGA CAC GTG GCA TGA TTC TAT |  | CGA CAC GTG GCA TGA TTC TAT |
| Lgm 4 | TTT TAG ATT TAG GGT TTC | Lgm 24 | GCC AC CAGA TCT TGC ATC GT |
|  | GTT CCT GGA GAT GTT CAG |  | TTG CAA GCT TGT CTC CTG ATT |
| Lgm 5 | GGCTGAGGAAGAGTTACGGG | Lgm 25 | AAG GAG GAG TAG AAG GAG |
|  | CCG GAT TGA GCC AAA CGA AC |  | GTT ATG AAC CAT GTG CTG |
| Lgm 6 | TGT CCC TTT GTT TTG GGC TT | Lgm 26 | CCG CTG GTA GTC CTA TGT CA |
|  | ACA CCC ATG GAT GGT GGA AGA |  | ATG TAC TCC GGA GAT TTG GAG A |
| Lgm 7 | GCT CCC ACC ATG TTT TGC TT | Lgm 27 | GCC ACT CCG ATG CTC AAG T |
|  | TGA GGT GGG CGT AAT TCA TGT |  | CCT AGA GGG CAA AAG GGG AAT |
| Lgm 8 | GCT TTA TTG TTT TTG GCG GGG | Lgm 28 | GTG CAC TCG ACA GAA GGT GG |
|  | CCC CTA ATG CCC TAG GCA AC |  | GTG TTC CGC ATT GGC TAG G |
| Lgm 9 | TTC GCG GGT TGA CTT CTT GA | Lgm 29 | AGC ACC AAA TTC GAC TCA ATG C |
|  | CAG CAC CTG AAC CAC TCA GA |  | AGG CAA CTC ATT CAT ATT CCA CA |
| Lgm 10 | ATA GCA GCT TTT CGG AGG GC | Lgm 30 | AGC AAT CCC AGT TAT GCA CAC |
|  | AGT CCG AAT TTC TCC TCC TCA TC |  | CGG CCA ATG TCG GCT GAT G |
| Lgm 11 | CGA CAC TGG TGT TGA AGG AC | Lgm 31 | TCT CGA AAC CAA GCG GGA C |
|  | ACG TCT GGA ACA CCG ATA CA |  | AGC TGC TCC TTA ATC CGA AC |
| Lgm 12 | AGGCGTATGAGTAGGTGTA | Lgm 32 | AAA TCT GCT TTT ACC ATC |
|  | GAG TAT TTC TCG GGC TTC |  | CTT ACC ACT TAA CCA CGA |
| Lgm 13 | TCC CAA TAA GTG CTT CGG CA | Lgm 33 | CCA TGT GCC AGG AAG GGT TA |
|  | TGG GTG GGT ATC ATC CTC GT |  | AGT AAC AGA TGT GAA ACC CAA CA |
| Lgm 14 | CGG TTA TGA ACC GTG TGC TG | Lgm 34 | TCA AAG GAC ATA AAT AAC |
|  | TGT GTG GAA TTG TGA GCG GA |  | AAC CCA CTA ACT AAT ATC |
| Lgm 15 | CGT TTG GGA TGG GGG TGT AG | Lgm 35 | AGT GAG AGA AAC TCA GAA ACT CCC |
|  | GCG TGA ATC GGG TCT ACA GG |  | CCA CTG GCA AGA TAT GCA ACA |
| Lgm 16 | GGT CCG AAT CGG TGA TGG AG | Lgm 36 | GGC ATA GTC AGT GCA GCT TG |
|  | CTC TCA ACC CGA TAA CCC CC |  | TGC TGC CTT TTT CGC CTC TA |
| Lgm 17 | GCA CCA TTG GAA GGG TGT GA | Lgm 37 | ACG ACA CGT GGC ATG ATT CTA T |
|  | CAG GCA TTT GAG CCG AAA CA |  | CGA GTC AGT GAG CGA GGA AG |
| Lgm 18 | CGT CGA GGG AGT AAG ATT CG | Lgm 38 | CCT CCC ACT TTC CAA CTC CC |
|  | GGT GCG GGT TAG AGG AAT GG |  | GCG ACC AAT ATC GGC TGA AG |
| Lgm 19 | GCC ACT GTC GTA ATA CAA GCA C | Lgm 39 | TTA CAA CAC GTG GCA TGA TTC T |
|  | ATT GGT GCG TTG GTC TTT GC |  | TGT GTG GAA TTG TGA GCG GA |
| Lgm 20 | ATT AGG CTA CCT CAT CTG | Lgm 40 | GTC GAC GAT TCC CTG GTT GG |
|  | TTC TGT AAG GGT TCT TCT |  | TTT ACT CCC TGG CAT GAG CG |

Lgm 1, Lgm 3, Lgm 6, Lgm 7, Lgm 8, Lgm 9, Lgm 10, Lgm 11, Lgm 13, Lgm 16, Lgm 23, Lgm 24, Lgm 26, Lgm 28, Lgm 29, Lgm 31 and Lgm 34 can amplify clear bands; Lgm 1、Lgm 6、Lgm 7、Lgm 10、Lgm 11、Lgm 13、Lgm 16、Lgm 26、Lgm 28、Lgm 29、Lgm 31、Lgm 34 have polymorphism.

Table S3 The coordinate points of *G. przewalskii* used for SDM models.

| species | x | y |
| --- | --- | --- |
| G.prz | 93.937 | 42.894 |
| G.prz | 91.383 | 43.333 |
| G.prz | 94.452 | 42.595 |
| G.prz | 81.633 | 41.85 |
| G.prz | 81.25 | 41.55 |
| G.prz | 79.067 | 40.55 |
| G.prz | 82.77 | 41.85 |
| G.prz | 75.01 | 39.68 |
| G.prz | 75.83 | 39.42 |
| G.prz | 84.23 | 41.77 |
| G.prz | 95.505 | 41.105 |
| G.prz | 94.719 | 39.669 |
| G.prz | 94.343 | 39.598 |
| G.prz | 99.462 | 40.322 |
| G.prz | 98.198 | 39.785 |
| G.prz | 96.786 | 39.9 |
| G.prz | 99.834 | 39.383 |
| G.prz | 96.983 | 40.247 |
| G.prz | 104.71 | 39.648 |
| G.prz | 108.5 | 41.67 |
| G.prz | 104.919 | 37.434 |
| G.prz | 86.984 | 42.271 |
| G.prz | 87.735 | 40.631 |
| G.prz | 90.365 | 44.544 |
| G.prz | 104.2 | 36.57 |
| G.prz | 105.2 | 37.48 |

Table S4 Climate variables used to fit niche model.

| **Variable** |  | **Source** |
| --- | --- | --- |
| Bio1 | Annual mean temperature | <http://www.worldclim.org/> |
| Bio2 | Mean diurnal temperature range | <http://www.worldclim.org/> |
| Bio4 | Temperature seasonality | <http://www.worldclim.org/> |
| Bio9 | Mean temperature of the driest quarter | <http://www.worldclim.org/> |
| Bio12 | Annual mean precipitation | <http://www.worldclim.org/> |
| Bio14 | Precipitation of the driest month | <http://www.worldclim.org/> |
| Bio15 | Precipitation seasonality | <http://www.worldclim.org/> |

Table S5 Genetic diversity among the loci of *G. przewalskii.*

| 位点  Locus | *N_A_* | *N_E_* | *H*o | *H_E_* | *F*is | *F*it | *F*st | *N*m |
| --- | --- | --- | --- | --- | --- | --- | --- | --- |
| Lgm 1 | 9 | 3.8227 | 0.7279 | 0.7411 | -0.1579 | 0.0142 | 0.1486 | 1.4324 |
| Lgm 7 | 8 | 4.6754 | 0.6838 | 0.7890 | -0.0583 | 0.1301 | 0.1781 | 1.1540 |
| Lgm 11 | 15 | 8.2979 | 0.5441 | 0.8827 | 0.1984 | 0.3813 | 0.2282 | 0.8454 |
| Lgm 13 | 12 | 3.3747 | 0.5556 | 0.7063 | -0.1787 | 0.2108 | 0.3304 | 0.5067 |
| Lgm 28 | 15 | 4.6398 | 0.3407 | 0.7874 | 0.4059 | 0.5647 | 0.2673 | 0.6854 |
| Lgm 29 | 10 | 2.1348 | 0.5221 | 0.5335 | -0.1281 | 0.0179 | 0.1294 | 1.6816 |
| Mean | 11.5 | 4.4909 | 0.5624 | 0.7400 | 0.0253 | 0.2371 | 0.2173 | 0.9004 |

*N*a mean number of alleles*, N*e mean number of effective alleles, *H*o mean observed heterozygosity, *H*e mean expected heterozygosity, *F* mean allelic fixation index, *N*m mean gene flow.

Table S6 The Nei's genetic identity and genetic distance of *G. przewalskii.*

| Population | WQ | SF | WS | BC1 | BC2 | KC | LT | HM1 | HM3 |
| --- | --- | --- | --- | --- | --- | --- | --- | --- | --- |
| WQ |  | 0.8586 | 0.7958 | 0.7136 | 0.7328 | 0.8475 | 0.8998 | 0.7118 | 0.7861 |
| SF | 0.1524 |  | 0.7963 | 0.7269 | 0.7009 | 0.8227 | 0.806 | 0.7746 | 0.8061 |
| WS | 0.2284 | 0.2277 |  | 0.8551 | 0.8248 | 0.9501 | 0.8588 | 0.6359 | 0.7474 |
| BC1 | 0.3375 | 0.319 | 0.1566 |  | 0.8475 | 0.8653 | 0.7947 | 0.6353 | 0.692 |
| BC2 | 0.3109 | 0.3554 | 0.1927 | 0.1655 |  | 0.8761 | 0.865 | 0.6309 | 0.6623 |
| KC | 0.1655 | 0.1951 | 0.0512 | 0.1447 | 0.1323 |  | 0.9239 | 0.6806 | 0.7493 |
| LT | 0.1055 | 0.2156 | 0.1522 | 0.2298 | 0.1451 | 0.0791 |  | 0.7057 | 0.7258 |
| HM1 | 0.34 | 0.2554 | 0.4527 | 0.4537 | 0.4605 | 0.3848 | 0.3486 |  | 0.7506 |
| HM3 | 0.2407 | 0.2155 | 0.2912 | 0.3682 | 0.412 | 0.2886 | 0.3205 | 0.2868 |  |
| AKS | 0.2147 | 0.1592 | 0.3211 | 0.3344 | 0.3145 | 0.3073 | 0.3092 | 0.3597 | 0.1424 |
| SB | 1.2536 | 1.1902 | 0.8453 | 0.7664 | 1.1498 | 0.9677 | 1.1163 | 0.9429 | 0.9348 |
| LY | 1.139 | 1.0525 | 0.8029 | 0.7238 | 1.0838 | 0.9218 | 0.9808 | 0.811 | 0.9075 |
| CM | 1.0392 | 1.1114 | 0.8828 | 0.7402 | 1.0302 | 0.9523 | 1.0879 | 0.9982 | 0.9001 |
| JYG | 0.2685 | 0.2238 | 0.3058 | 0.3382 | 0.3471 | 0.307 | 0.3122 | 0.167 | 0.1266 |
| GT | 0.1675 | 0.1209 | 0.2295 | 0.3172 | 0.357 | 0.232 | 0.2126 | 0.1971 | 0.1299 |
| ZW | 0.745 | 0.7415 | 0.8213 | 0.686 | 0.9819 | 0.8248 | 0.8093 | 0.7286 | 0.7772 |
| AZQ | 1.1557 | 1.282 | 0.8956 | 0.8723 | 1.2196 | 1.0083 | 1.0116 | 0.9901 | 0.8562 |

| Population | AKS | SB | LY | CM | JYG | GT | ZW | AZQ |
| --- | --- | --- | --- | --- | --- | --- | --- | --- |
| WQ | 0.8068 | 0.2855 | 0.3201 | 0.3537 | 0.7645 | 0.8458 | 0.4748 | 0.3149 |
| SF | 0.8528 | 0.3042 | 0.3491 | 0.3291 | 0.7994 | 0.8861 | 0.4764 | 0.2775 |
| WS | 0.7254 | 0.4294 | 0.448 | 0.4136 | 0.7365 | 0.7949 | 0.4398 | 0.4084 |
| BC1 | 0.7158 | 0.4647 | 0.4849 | 0.477 | 0.713 | 0.7282 | 0.5036 | 0.418 |
| BC2 | 0.7301 | 0.3167 | 0.3383 | 0.3569 | 0.7068 | 0.6998 | 0.3746 | 0.2954 |
| KC | 0.7354 | 0.38 | 0.3978 | 0.3858 | 0.7357 | 0.7929 | 0.4383 | 0.3648 |
| LT | 0.7341 | 0.3275 | 0.375 | 0.3369 | 0.7319 | 0.8085 | 0.4452 | 0.3636 |
| HM1 | 0.6979 | 0.3895 | 0.4444 | 0.3685 | 0.8462 | 0.8211 | 0.4826 | 0.3715 |
| HM3 | 0.8673 | 0.3927 | 0.4035 | 0.4065 | 0.881 | 0.8782 | 0.4597 | 0.4248 |
| AKS |  | 0.278 | 0.2772 | 0.3845 | 0.8753 | 0.8774 | 0.3923 | 0.2288 |
| SB | 1.2802 |  | 0.8282 | 0.6717 | 0.363 | 0.3596 | 0.4911 | 0.7591 |
| LY | 1.2831 | 0.1885 |  | 0.5289 | 0.4138 | 0.4335 | 0.6487 | 0.8986 |
| CM | 0.9559 | 0.3979 | 0.637 |  | 0.4105 | 0.4187 | 0.6431 | 0.5081 |
| JYG | 0.1332 | 1.0133 | 0.8823 | 0.8903 |  | 0.9195 | 0.4512 | 0.3661 |
| GT | 0.1308 | 1.0229 | 0.8359 | 0.8706 | 0.0839 |  | 0.4892 | 0.3913 |
| ZW | 0.9357 | 0.7112 | 0.4328 | 0.4415 | 0.7957 | 0.7149 |  | 0.5883 |
| AZQ | 1.4749 | 0.2756 | 0.1069 | 0.677 | 1.0048 | 0.9382 | 0.5305 |  |

Nei's genetic identity (above diagonal) and genetic distance (below diagonal).
